# Gene Expression Profile of Glioblastoma Peritumoral Tissue: An Ex Vivo Study

**DOI:** 10.1371/journal.pone.0057145

**Published:** 2013-03-05

**Authors:** Annunziato Mangiola, Nathalie Saulnier, Pasquale De Bonis, Daniela Orteschi, Gigliola Sica, Gina Lama, Benedetta Ludovica Pettorini, Giovanni Sabatino, Marcella Zollino, Libero Lauriola, Anna Colabianchi, Gabriella Proietti, Gyula Kovacs, Giulio Maira, Carmelo Anile

**Affiliations:** 1 Institute of Neurosurgery, Faculty of Medicine, Catholic University of Rome, Rome, Italy; 2 Institute of Human Anatomy, Faculty of Medicine, Catholic University of Rome, Rome, Italy; 3 Institute of Pathology, Faculty of Medicine, Catholic University of Rome, Rome, Italy; 4 Institute of Histology and Embryology, Faculty of Medicine, Catholic University of Rome, Rome, Italy; 5 Institute of Genetics, Faculty of Medicine, Catholic University of Rome, Rome, Italy; 6 Medical Faculty, Ruprecht Karls University, Heidelberg, Germany; Catholic University Medical School, Italy

## Abstract

The gene expression pattern of glioblastoma (GBM) is well documented but the expression profile of brain adjacent to tumor is not yet analysed. This may help to understand the oncogenic pathway of GBM development. We have established the genome-wide expression profiles of samples isolated from GBM tumor mass, white matter adjacent to tumor (apparently free of tumor cells), and white matter controls by using the Affymetrix HG-U133 arrays. Array-CGH (aCGH) was also performed to detect genomic alterations. Among genes dysregulated in peritumoral white matter, 15 were over-expressed, while 42 were down-regulated when compared to white matter controls. A similar expression profile was detected in GBM cells. Growth, proliferation and cell motility/adhesion-associated genes were up-regulated while genes involved in neurogenesis were down-regulated. Furthermore, several tumor suppressor genes along with the *KLRC1* (a member of natural killer receptor) were also down-regulated in the peritumoral brain tissue. Several mosaic genomic lesions were detected by aCGH, mostly in tumor samples and several GBM-associated mosaic genomic lesions were also present in the peritumoral brain tissue, with a similar mosaicism pattern. Our data could be explained by a dilution of genes expressed from tumor cells infiltrating the peritumour tissue. Alternatively, these findings could be substained by a relevant amount of “apparently normal” cells presenting a gene profile compatible with a precancerous state or even “quiescent” cancer cells. Otherwise, the recurrent tumor may arise from both infiltrating tumor cells and from an interaction and recruitment of apparently normal cells in the peritumor tissue by infiltrating tumor cells.

## Introduction

Glioblastoma (GBM) is the most common malignant tumor of the brain. GBM rapidly proliferates and invades the central nervous system. Due to his invasive characteristics, the prognosis of GBM patients is very poor, despite of the treatment that currently consists of surgical resection followed by radiotherapy plus concomitant and adjuvant temozolomide [1]. Targeted therapies have been introduced, based on information obtained from molecular studies of the tumor tissue (usually shown at MRI as an enhanced lesion, ET) [2]. However, no clear survival benefit has been demonstrated, probably because tumor tissue represents the last step of tumorigenesis, involving some alterations allowing tumor-cells to survive. Since recurrence in peritumoral tissue occurs in about 95% of patients [3], getting a deeper insight into the biology of the brain adjacent to-tumor (BAT) is of great interest. It has been demonstrated that the expression of a series of elements and amino-acids is altered in the BAT [4], [5], [6], [7], [8]. It has been shown that even in the absence of tumor cells, kinases involved in cell proliferation, migration and apoptosis are expressed in BAT together with molecules linked to stemness, invasion and neo-angiogenesis, which might indicate that BAT is undergoing transformation [9], [10], [11]. To support this hypothesis, we carried out a gene expression profile and a genomic analysis of BAT to detect alterations that may indicate the appearance of neoplastic features. To achieve our aim, we have compared the gene expression profile of tissue samples from ET, BAT, and normal white matter (CTRL) and have analyzed the BAT with an Array-CGH to detect genomic alterations.

## Methods

### Patients and Specimens

**Figure 1:**
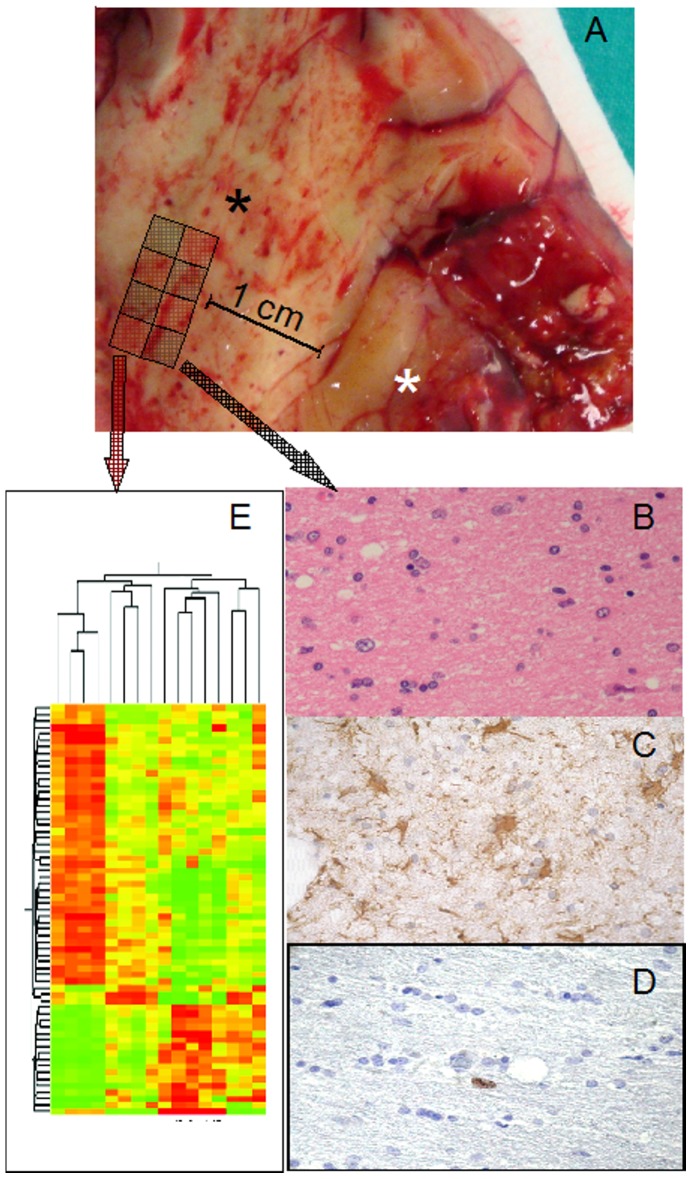
Tissue sampling. (A) Tissue specimen opened in a book-wise fashion before sampling; white asterisk: tumor; black asterisk: BAT. Sampling-grid: each tissue specimen was generally divided into eight parts, and BAT samples for molecular analyses were contiguous to those used for histology; in this way, a higher probability of homogeneity between samples used for histology and gene expression analysis, quantitative real-time PCR, western blot analysis and array-CGH was obtained. (B) H&E staining of BAT with absence of morphologically neoplastic cells. (C) GFAP staining, showing reactive astrocytes, with stellar morphology. (D) Ki67/MIB-1 was always <1%. (E) Gene-microarray analysis.

From January 2006 to December 2007, 60 adults were operated for primary GBM at our Institute. We selected 11 patients with tumor location far from eloquent areas. Among them, 5 patients did not present tumor cells in BAT and were included in the study. Tumor was removed with wide tumor-free resection margins of 1–2 cm. One part of the ET (without areas of necrosis) was immediately fixed in 10% neutral buffered formalin for histological analysis and the second one was immediately frozen on dry ice for molecular analysis. BAT specimens were obtained using a sampling-grid (Figure1A). CTRL specimens were derived from 4 patients at the same ages operated for deep cavernomas with radiological signs of recent bleeding. These samples were used as controls in molecular analyses.

Ethics statement. All patients provided written consent to use their specimens for research purposes, none of them was identifiable. The study was approved by the local ethics committee (Catholic University Ethics Committee, Rome). The ethical principles of the declaration of Helsinki were strictly followed.

### Histopathology

All histological samples were reviewed by a board-certified neuropathologist (LL) and all tumours were classified as glioblastoma (WHO IV). Multiple levels of each paraffin block of samples used for research purposes (ET, BAT and CTRL) were thoroughly examined. No tumor cells were seen in BAT samples used in this study.

### Gene Expression Analysis

For global gene expression analysis we used 4 CTRL samples, 5 ET samples (ET1, ET2, ET3, ET4, ET5), and 7 BAT samples (BAT1, BAT3, BAT5; in two patient samples were taken from two different peritumoral areas: BAT2, BAT2R; BAT4, BAT4R). Total RNA was extracted using Trizol Reagent followed by clean-up and DNase digestion on an RNeasy spin column. RNA was quantified by UV spectrophotometer and quality was assessed on agarose gel. RNA was processed for use on the Affymetrix Human Expression HG-U133A arrays (Affymetrix, Santa Clara, CA) according to manufacturer’s instructions. Briefly, 2.5 µg of each RNA was converted into double-strand cDNA using a T7-(dT)24 primer. cDNA was used as template to generate biotinylated cRNA during an in vitro transcription step. Labeled cRNA was purified, chemically fragmented and 15 µg were hybridized on the array for 16 h at 45°C in a rotisserie oven set at 60 rpm. The arrays were then stained in the Affymetrix Fluidic Station and scanned twice using the Agilent Gene Array scanner 2500.

The expression data were generated by Affymetrix microarray suite 5.0 software and loaded into GeneSpring Expression Analysis version 7.3 software. Raw intensities from each chip were normalized using the GC-RMA method completed by an additional normalization to the median for each gene. Data were filtered to eliminate genes displaying an averaged intensity inferior to the global array background.

Datasets were then assigned to the three experimental groups: CTRL, ET, and BAT, and the averaged log2 intensities of biological replicates were used for further analysis. Homogeneity of sample groups was verified by analysis of the principal component (PCA). Pre-filtered data were submitted to statistical analysis (t-test; P<0.05 with FDR correction) to identify genes differentially expressed between ET and BAT samples, and between BAT and CTRL. Genes with a fold change of gene expression >2 between CTRL and BAT samples were subjected to hierarchical clustering using Pearson correlation coefficient. Results have been deposited in NCBI Gene Expression Omnibus http://www.ncbi.nlm.nih.gov/projects/geo/ (accession number: GSE13276).

### Quantitative Real-time PCR

About 1.5 µg of total RNA were reverse-transcribed by SuperScript III, using random hexamer primers (Invitrogen, Carlsbad, CA, USA). Quantitative real-time PCR (qPCR) was carried out with LightCycler technology (Roche Molecular Biochemicals, Indianapolis, IN, USA). Oligonucleotide primers (Table 1) were designed using Primer3 software (http://frodo.wi.mit.edu/). Cycling conditions: 95°C for 10 minutes, 40 cycles of 95°C for 10 seconds, 58°C for 7 seconds, 72°C for 8 seconds, followed by a melt curve analysis immediately begun to rule out synthesis of unspecific products. Crossing points (Cp) of real-time-PCR curves were determined by the Light cycler software using the second derivative maximum method. For each target, two independent amplifications were performed and the mean value was used for further analysis. The 2−ΔΔCt method was applied to calculate fold changes in the expression levels of the genes. Statistical analysis was performed using t-test.

**Table 1:** Primer sequences.

Gene	Primer forward (5′–3′)	Primer reverse (5′–3′)
*TAZ*	CAGCAATGTGGATGAGATGG	TCATTGAAGAGGGGGATCAG
*KLRC1*	CATCCTCATGGATTGGTGTG	GATCCACACTGGGCTGATTT
*EGFR*	TGCTGGATGATAGACGCAGA	GGCACGGTAGAAGTTGGAGT
*IGFBP5*	CAGGGGTCTGGTCTCTTTGA	GTGGAGTGAGGCACGAATCT
*SRP1*	TGAGGACGAGGTGGATGTTA	CCCCTGTAGCCATACTTTGC
*USH1C*	GAAGAAGACTGGGGCTCAAA	AAATCCTGCTCTCCCTGCTC
*ID3*	GGAGCTTTTGCCACTGACTC	CAGGAAGGGATTTGGTGAAG
*18S ribosomal*	Qiagen Hs_RRN18S_1_SG QuantiTect_Primer_Assay (QT00199367)	

### Western-blot Analysis

The presence of KLRC1 (killer cell lectin-like receptor sub family C, member1) in 3 independent specimens of BAT and 3 CTRL samples was evaluated by western-blot analysis. Forty µg of proteins were resolved by 12% SDS-PAGE and transferred to PVDF membrane. Non specific binding was blocked with 5% nonfat dry milk for 1 h. The blots were probed with a 1∶500 dilution of mouse anti-human KLRC1 antibody (Abnova Corporation.) or a 1∶10.000 dilution of mouse anti-human β-actin, overnight at 4°C. Biotinylated anti-mouse IgG (dilution 1∶1000; Vector Laboratories, Burlingame, CA, USA) was used as secondary antibody for 1 h at RT. After washes in PBS containing 0.1% Tween-20, membranes were incubated for 30 min at RT with Vectastain Elite ABC reagent (Vector Laboratories), and antibody reactivity was visualized by incubation with diaminobenzidine.

### Array-CGH

DNA from 3 frozen ET samples (ET1, ET2 and ET3) and 3 matched BAT samples (BAT1, BAT2R and BAT3) was extracted using QIAamp DNA Mini Kit (QIAGEN, Hilden, Germany). The rest of frozen tissue of BAT1, BAT2R and BAT3 samples was then evaluated in frozen sections and no tumor cells were identified.

Oligonucleotide aCGH was performed using the Agilent Human Genome CGH microarray 4×44K, (Agilent Technologies Santa Clara, CA, USA), with an average resolution of 75 kb, following the manufacturer’s instructions. Each sample was paired with a sex-matched commercial control DNA (Promega, Madison, WI, USA), and hybridized twice in a dye-swap experiment, in order to minimize false positive calls. The arrays were analyzed using GenePix 4000B scanner (Axon, Union City, CA, USA) and Feature Extraction V.9.5.1 software. A graphical overview of the results was obtained using CGH Analytics V.3.5.14 software. Combined dye-swapped experiments were analyzed with ADM-2 algorithm at 3 different thresholds: 4, 5 and 6, with Centralization and Fuzzy Zero corrections turned on, with the purpose of detecting low grade mosaicisms. The percentage of abnormal cells was inferred using the formula proposed by Valli et al. [12].

### Immunohistology

Both immunohistochemical and immunofluorescence analyses were performed in paraffin-embedded tissue sections (5 µm thick) from ET and BAT. All samples were deparaffinized and rehydrated. Immunohistochemical and immunofluorescence analyses were performed considering the results of the gene expression analyses. In particular, the expression of some proteins whose genes were over-expressed in the BAT compared with CTRL was analyzed. Moreover, immunofluorescence analysis for CD133 protein was performed.

For immunohistochemistry, after the endogen peroxidase blocking, sections were incubated with monoclonal anti-human GFAP (1∶100; clone 273807; R&D SYSTEMS, Minneapolis, MN, USA), anti-human Ki-67 (1∶100; clone MIB-1; Dako, Glostrup, Denmark) antibodies or with a polyclonal anti-human TAZ antibody (1∶60; LifeSpan Biosciences, Seattle, WA, USA) overnight at 4°C. Subsequently, slides were incubated with a HRP/Fab polymer conjugate (SuperPicTure Polymer Detection Kit, Invitrogen, Camarillo, CA, USA). The immunostaining for Epidermal Growth Factor Receptor (EGFR) and CD99 was performed using the monoclonal antibodies anti- human EGFR (1∶100; Clone E30; Dako) and CD99 (1∶100; clone 12E7; Dako) on an autostainer (Dako Autostainer Plus Link, Dako). The antigen retrieval was performed using pronase digestion (ProTaqs® Pronase Digest, Germany) and En Vision™ Flex Target Retrieval Solution High pH (Dako) for EGFR and CD99, respectively.

Immunopositive cells were visualized by brown DAB (Vector Laboratories, Inc., Burlingame, CA, USA) staining. The nuclei were lightly counterstained with Mayer’s hematoxylin.

Tonsil sections were used as positive controls for EGFR and CD99 expression.

For CD133 immunofluorescence analysis on ET and BAT, the sections were incubated for 12 h at 4°C with the polyclonal anti-human CD133 (1∶50; Spring Bioscience, CA, USA) and were then treated with the secondary antibody (goat anti-rabbit Alexa Fluor 488; 1∶200; Invitrogen) for 1 h at room temperature (RT).

For double-labeling immunofluorescence analysis, histological sections were incubated for 20 h at 4°C with the anti-GFAP and TAZ antibodies as described above. The next day, the slides were incubated with a mixture of the following secondary antibodies: goat anti-rabbit Alexa Fluor 488 (1∶250; Invitrogen) and red fluorescent cyanine donkey anti-mouse Cy3 (1∶200; Jackson Immunoresearch Laboratories) for 2 h at RT. The sections were coverslipped with Vectashield Mounting Medium with DAPI (Vector Laboratories) and examined with a confocal laser scanning system (TCS-SP2, Leica Microsystems, GmbH, Wetzlar, Germany) equipped with an Ar/ArKr laser and a HeNe laser for 488 nm and 543 nm excitation, respectively. For each analyzed field, Z-stack series of 4–5 µm-thick were acquired as images (1024×1024 pixels), recorded at intervals of 0.20 µm, and then projection images were created and processed using the Leica software. MDA-MB-231, a breast cancer cell line expressing moderate TAZ levels, was used as positive control for TAZ expression [13]. In each experiment, negative controls without the primary antibody were included to check for nonspecific staining.

## Results

### Histopathology

The CTRL samples displayed a variable degree of gliosis due to bleeding of the cavernomas and subsequent edema, as it was indicated by the presence of GFAP-reactive astrocytes, with a characteristic dendritic morphology and abundant eosinophilic cytoplasm and with large eccentric nuclei (data not shown). The number of Ki67/MIB1 positive cells was lower than 1% (data not shown).

In the ET samples, the number of Ki67/MIB1 positive cells varied between 10% and 60% (data not shown).

### Microarray Analysis

**Figure 2:**
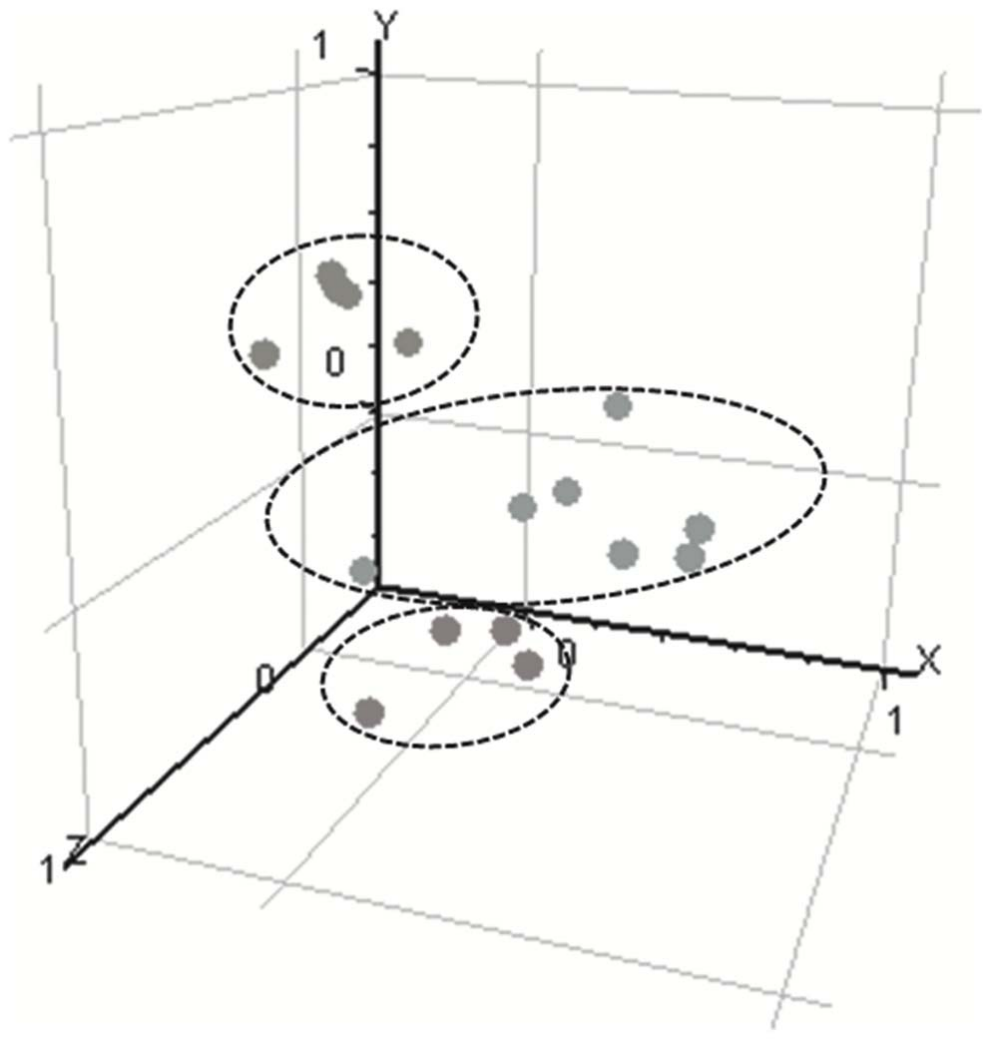
PCA 3D view for gene expression profiles of samples of the three experimental conditions (red: CTRL; yellow: ET; blue: BAT). Every dot represents a sample. PCA was based on log^2^ ratios and the expression profiles were performed across the 14,500 genes of the human HG-U133A array. The first 3 principal components are plotted. PCA representation shows samples segregation according to their tissue origin.

Genome-wide expression profiles of 5 ET samples, 7 BAT samples from 5 patients, and 4 CTRL white matter biopsies were analysed. The representation of the samples in a three-dimensional (3D) space clearly distinguished 3 groups corresponding to the 3 tissues (Figure 2). The CTRL and BAT samples were pooled in 2 distinct groups.

### Differential Gene Expression between ET and BAT

Molecular analysis of ET and BAT samples showed significant changes in the expression of 1,323 genes. Genes with at least 10-fold difference in the expression level were further analyzed (Table 2). Twenty genes were over-expressed in ET when compared to BAT, while 45 genes were down-regulated. The expression of the angiogenesis-related genes *VEGF* and *ANGPT2* was increased in ET. Genes associated with cell growth (*IGFBP2*, *GAP43*), along with the cell cycle activator *CKS2*, were over-expressed. Most of the highly up-regulated genes encoded proteins associated with the extracellular matrix formation, including *COL4A1, COL4A2, COL1A1, COL3A1, COL1A2*. The 45 genes showing significantly decreased expression in ET were more heterogeneous with respect to the functions of their gene products. The majority of these genes were involved in the development of the nervous system (*MOG, RAPGEF5, GRM3, SH3GL3, NINJ2, UGT8, MOBP, MBP*).

**Table 2:** Selection of the genes significantly different between ET and BAT with a 10*-*fold difference in expression levels.

Genebank	Description	Symbol	FC	Corrected p-value
**Genes down-regulated in ET compared to BAT**				
***Central nervous system development***				
NM_006501	myelin-associated oligodendrocyte basic protein	MOBP	−34.4	0.006
NM_003027	SH3-domain GRB2-like 3	SH3GL3	−31.7	0.022
NM_002385	myelin basic protein	MBP	−29.2	0.029
NM_002433	myelin oligodendrocyte glycoprotein	MOG	−24.2	0.018
NM_016533	ninjurin 2	NINJ2	−16	0.039
NM_000840	glutamate receptor, metabotropic 3	GRM3	−13.8	0.035
NM_003360	UDP glycosyltransferase 8	UGT8	−12.9	0.004
NM_012294	Rap guanine nucleotide exchange factor 5	RAPGEF5	−12	0.03
***Signal transduction***				
NM_002371	mal, T-cell differentiation protein	MAL	−30.6	0.017
AL524520	G protein-coupled receptor 49	GPR49	−12.7	0.045
T16257	G protein-coupled receptor 37	GPR37	−12.6	
NM_005709	Usher syndrome 1C	USH1C	−10	0.014
***Cell adhesion/motility***				
L35594	autotaxin	ATX	−22.3	0.037
X98405	myelin associated glycoprotein	MAG	−18.7	0.018
U88870	peanut-like 2	PNUTL2	−14.7	0.042
NM_016950	sparc/osteonectin, cwcv and kazal-like domains proteoglycan (testican) 3	SPOCK3	−11	0.006
NM_003628	plakophilin 4	PKP4	−10.8	0.017
***Transport***				
NM_001063	transferrin	TF	−15	0.004
NM_018478	dysbindin	DBNDD2	−12.4	0.026
NM_012128	chloride channel, calcium activated, family member 4	CLCA4	−11.8	0.004
NM_007168	ATP-binding cassette, sub-family A member 8	ABCA8	−11.3	0.007
BC000585	solute carrier organic anion transporter family, member 3A1	SLCO3A1	−10	0.022
***Protein metabolism***				
NM_002774	kallikrein 6	KLK6	−32	0.004
NM_002570	paired basic amino acid cleaving system 4	PACE4	−28.5	0.043
NM_000049	aspartoacylase	ASPA	−24.2	0.032
NM_004476	folate hydrolase 1	FOLH1	−18.5	0.031
NM_004616	transmembrane 4 superfamily member 3	TM4SF3	−10.8	0.005
***Transcription regulation***				
NM_014682	suppression of tumorigenicity 18	ST18	−30	0.028
NM_014717	zinc finger protein 536	ZNF536	−16.3	0.03
NM_013279	chromosome 11 open reading frame 9	C11orf9	−10.7	0.017
***Cytoskeleton organization***				
AU157109	KIAA1598 protein	KIAA1598	−14.8	0.048
AW242297	microtubule-associated protein 7	MAP7	−13.2	0.037
***Other processes***				
AA191573	synaptojanin 2	SYNJ2	−35.4	0.022
AB032981	PAIP2B HGNC binding protein interacting protein 2B	PAIP2B	−27	0.047
AB007880	KIAA0420 gene product	KIAA0420	−22.5	0.039
AI803302	LIM domain binding 3	LDB3	−20.4	0.028
U56725	Human heat shock protein mRNA,	HSPA2	−20	0.038
BC003169	calpain 3, (p94)	CAPN3	−18.2	0.027
NM_000954	prostaglandin D2 synthase 21 kDa (brain)	PTGDS	−17.6	0.026
NM_024306	fatty acid 2-hydroxylase	FA2H	−13.5	0.038
NM_022126	phospholysine phosphohistidine inorganic pyrophosphate phosphatase	LHPP	−13	0.041
AF318616	synaptojanin 2	SYNJ2	−12	0.027
NM_014210	ecotropic viral integration site 2A	EVI2A	−11.7	0.039
NM_024897	progestin and adipoQ receptor family member VI	PAQR6	−11	0.002
NM_003657	breast carcinoma amplified sequence 1	BCAS1	−10.6	0.015
Genes over-expressed in ET compared to BAT				
***Immune system***				
NM_002852	pentaxin-related gene, rapidly induced by IL-1 beta	PTX3	18.2	0.028
NM_012072	complement component 1,q subcomponent, receptor 1	C1QR1	16.2	0.039
***Extracellular matrix formation***				
NM_000089	collagen, type I, alpha 2	COL1A2	26.8	0.014
X05610	collagen, type IV, alpha 2	COL4A2	13.9	0.017
K01228	collagen, type I, alpha 1	COL1A1	13.0	0.018
NM_001845	collagen, type IV, alpha 1	COL4A1	11.8	0.014
NM_000900	matrix Gla protein	MGP	10.4	0.002
X02761	fibronectin 1	FN1	10.2	0.03
AF130082	collagen, type III, alpha 1	COL3A1	10.0	0.008
***Angiogenesis***				
AF022375	vascular endothelial growth factor	VEGF	16.0	0.032
NM_001147	angiopoietin 2	ANGPT2	10.4	0.004
***Cell cycle***				
NM_001827	CDC28 protein kinase regulatory subunit 2	CKS2	12.6	0.017
***Cell growth & proliferation***				
NM_000597	insulin-like growth factor binding protein 2, 36 kDa	IGFBP2	35.8	0.017
NM_003254	tissue inhibitor of metalloproteinase 1	TIMP1	16.6	0.037
NM_002045	growth associated protein 43	GAP43	12.9	0.039
***Other processes***				
NM_000358	transforming growth factor, beta-induced, 68 kDa	TGFBI	26.6	0.026
BC001886	ribonucleotide reductase M2 polypeptide	RRM2	21.1	0.022
NM_014736	KIAA0101 gene product	KIAA0101	13.3	0.046
BG435404	ADP-ribosylation factor-like 7	ARL7	10.7	0.006
AI082078	actinin, alpha 1	ACTN1	10.2	0.015

### Differential Gene Expression between BAT and CTRL

**Figure 3:**
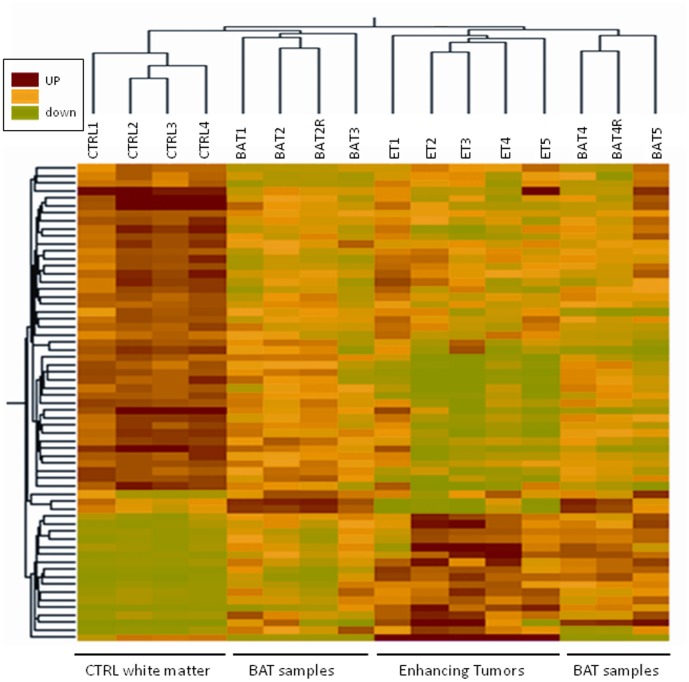
Hierarchical cluster analysis. Hierarchical cluster analysis based on expression of 63 transcripts (57 genes) that differed between BAT and CTRL samples (P<0.05) and had greater than 2-fold change between the means of the two tissue types. Each row represents a specific transcript; each column represents a tissue sample harvested from independent patients (R:technical replicate).

Statistical analysis associated with a threshold approach (cut-off: 2) resulted in 57 genes showing significantly different expression in BAT and CTRL. This dataset was submitted to hierarchical clustering to determine the gene expression pattern in all tissue types, including also ET specimens (Figure 3).

Molecular profiling showed 15 genes over-expressed and 42 genes down-regulated in BAT (Table 3). Genes belonging to 2 main relevant biological processes were particularly deregulated in BAT. In fact, genes associated with growth and proliferation (*CSRP2, TAZ, ID3, DTNA*) and cell motility/adhesion (*HIST2H2AA, EGFR, IGFBP5, VCAM1, CD99*) were up-regulated while genes involved in neurogenesis (*SYNJ1, NBEA, SERPINI1, CNTNAP2, RELN*) were largely down-regulated in BAT. Several tumour suppressor genes (*BAI3, PEG3, PRDM2, RB1CC1*) along with the natural killer receptor *KLRC1* were also down regulated in BAT.

**Table 3:** Selection of the genes significantly different between BAT samples and control white matter samples, with a 2*-*fold difference in expression levels.

Genebank	Description	Symbol	FC	Corrected *p-value*
**Genes up-regulated in BAT compared to CTRL**				
***Cell growth and proliferation***				
AW157070	epidermal growth factor receptor	EGFR	8.0	0.0389
AW007532	Homo sapiens cDNA clone IMAGE:2500861	IGFBP5	2.8	0.0253
NM_001321	cysteine and glycine-rich protein 2	CSRP2	2.4	0.0314
NM_001392	Homo sapiens dystrobrevin, alpha	DTNA	2.4	0.0295
NM_002167	inhibitor of DNA binding 3	ID3	3.3	0.0468
AI313324	histone H2A.1	HIST2H2AA	2.6	0.0468
***Cell adhesion/motility***				
NM_001078	vascular cell adhesion molecule 1	VCAM1	3.4	0.0159
U82164	CD99 antigen	CD99	2.7	0.0218
NM_005709	Usher syndrome 1C	USH1C	2.6	0.0192
NM_021077	neuromedin B	NMB	2.3	0.0468
BF674349	transcriptional co-activator with PDZ-binding motif	TAZ	2.7	0.0468
AU157932	palladin	PALLD	2.3	0.0256
***Apoptosis***				
NM_005460	synuclein, alpha interacting protein	SNCAIP	2.8	0.05
***Unknown function***				
AU154455	Homo sapiens cDNA clone NT2RP4001145	T1A-2	2.4	0.0314
NM_022074	FLJ22794 protein	FLJ22794	2.1	0.0192
**Genes down-regulated in BAT compared to CTRL**				
***Angiogenesis***				
NM_001704	brain-specific angiogenesis inhibitor 3	BAI3	−2.8	0.0468
***Transcription***				
AF208967	paternally expressed 3	PEG3	−5.6	0.0159
AI810712	hepatic leukemia factor	HLF	−5.5	0.0496
NM_004538	nucleosome assembly protein 1-like 3	NAP1L3	−3.3	0.0496
AL136629	TSPY-like	TSPYL1	−3.2	0.0468
AV721430	transcription factor 7-like 2	TCF7L2	−2.3	0.0496
AI096375	KIAA1750 protein	TSPYL5	−2.3	0.0168
NM_012231	PR domain containing 2, with ZNF domain	PRDM2	−2.5	0.0496
AA488899	protein associated with Myc	MYCBP2	−2.0	0.00518
BG402105	RB1-inducible coiled-coil 1	RB1CC1	−2.5	0.0192
Z98884	calmodulin binding transcription activator 1	CAMTA1	−2.6	0.0468
AB020663	Dmx-like 2	DMXL2	−2.0	0.0496
AL050331	Human DNA sequence from clone RP3-486I3 on chromosome 6q22.1–22.3	TSPYL4	−2.2	0.0314
***Signal transduction***				
NM_002738	protein kinase C, beta 1	PRKCB1	−11.3	0.0496
NM_007023	RAP guanine-nucleotide-exchange factor 4	RAPGEF4	−7.0	0.0496
NM_000807	gamma-aminobutyric acid (GABA) A receptor, alpha 2	GABRA2	−4.4	0.0158
NM_015678	neurobeachin	NBEA	−3.5	0.0496
AB020717	synaptojanin 1	SYNJ1	−2.9	0.0468
BC000498	aspartate aminotransferase 1	GOT1	−2.7	0.0496
AB007896	putative L-type neutral amino acid transporter	KIAA0436	−2.6	0.0159
NM_012093	adenylate kinase 5	AK5	−2.5	0.0468
L39833	potassium voltage-gated channel, shaker-related subfamily, beta member 1	KCNAB1	−2.4	0.0496
NM_014710	G protein-coupled receptor associated sorting protein	GASP	−2.2	0.0496
NM_014247	Rap guanine nucleotide exchange factor 2	RAPGEF2	−2.1	0.0131
AF052117	chloride channel 4	CLCN4	−2.1	0.0468
AB002390	lysosomal apyrase-like 1	LYSAL1	−2.0	0.0302
***Extracellular matrix remodelling***				
NM_000202	iduronate 2-sulfatase	IDS	−3.0	0.0496
AU144167	collagen, type III, alpha 1	COL3A1	−2.7	0.0468
***Growth***				
NM_020988	guanine nucleotide binding protein alpha activating activity polypeptide O	GNAO1	−2.1	0.0192
NM_000345	synuclein, alpha	SNCA	−2.1	0.0496
NM_005025	serine proteinase inhibitor	SERPINI1	−2.0	0.0192
***Adhesion***				
AW190070	ATPase, Ca++ transporting, cardiac muscle, slow twitch 2	ATP2A2	−2.5	0.0496
AU144598	contactin associated protein-like 2	CNTNAP2	−2.4	0.0453
NM_005045	reelin	RELN	−2.2	0.035
***Cytoskeleton***				
AB011133	microtubule associated serine/threonine kinase 3	MAST3	−2.2	0.0158
***Defense***				
NM_002260	killer cell lectin-like receptor subfamily C, member 1	KLRC1	−5.0	0.0125
***Ubiquitin cycle***				
AV726900	Ring finger protein 20	RNF20	−2.5	0.00518
AL031178	F-box protein 9	FBXO9	−2.2	0.0496
NM_018422	pleckstrin and Sec7 domain containing 3	PSD3	−2.5	0.0314
***Unknown***				
AL050136	Homo sapiens mRNA; cDNA DKFZp586L141		−2.2	0.0352
NM_014951	Zinc finger protein 365	ZNF365	−2.4	0.0192
AF063591	CD200 molecule	CD200	−2.2	0.0159

### Validation of Gene Expression by RT-PCR

**Figure 4:**
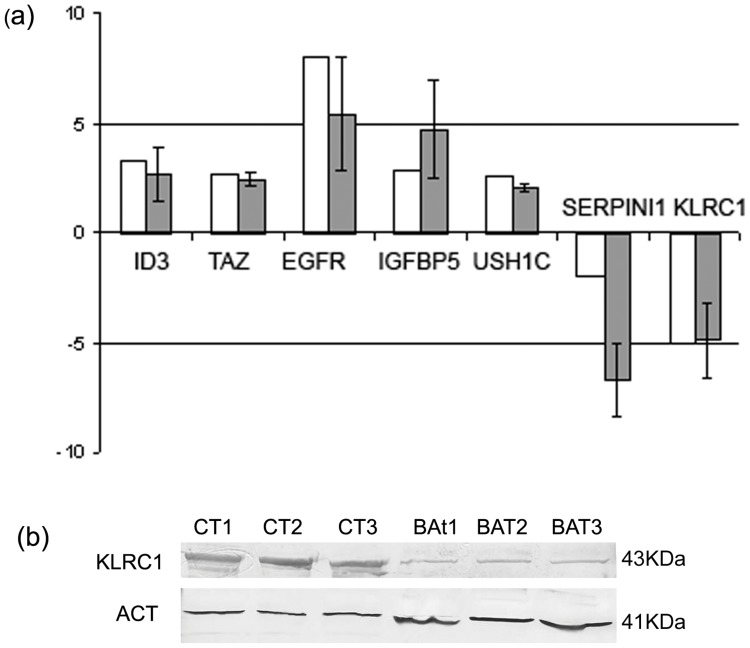
Validation of gene-expression. (a) qPCR validation of a subset of genes differentially expressed between BAT and CTRL. White bars represent the fold change in the expression level between BAT and CTRL as indicated by microarray analysis, while grey bars represent the mean fold change of gene expression calculated by qPCR method. The expression of each gene was normalized to that of 18S ribosomal RNA in the same sample and fold change represents the mean signal of 5 independent samples. Each grey bar is the mean ± SEM of duplicate determinations for each gene in biological replicates. *P*-values (t-test) for ID3, TAZ, EGFR, IGFBP5, USH1C, SERPINI1, KLRC1 were 0.043; 0.12; 0.09; 0.02; 0.005; 0.0004; 0.003; respectively. (b) KLRC1 western blot analysis in BAT and CTRL white matter samples. β-actin protein level was used as an endogenous control for loading.

A subset of 7 genes was selected for further analysis by qPCR (Figure 4a). The 2−ΔΔCt method was used to calculate fold changes in the expression levels of the selected genes in the comparison BAT vs CTRL samples. The qPCR results showed that the expression levels of *ID3, TAZ, EGFR, IGFBP5, USH1C* were increased in BAT samples, while the expression of *SERPINI1* and *KLRC1* was decreased. These results supported the validity of microarray results. KLRC1 was selected to assess differences in protein levels between BAT and CTRL samples by the means of western blot analysis (Figure 4b). KLRC1 was detected in all CTRL specimens, while a very weak signal was detectable in the BAT samples.

### Array CGH

A large number of mosaic genomic lesions were detected by aCGH in tumor samples. Some of these alterations, such as del (1p36), del (2p21), +7, del (6q27q29)/−6, *EGFR* (7p11.2), *MDM2* (12q15) and *CDK4* (12q14.3) amplification, −10, amplification of 15q24.1, del (17p13)/−17, −19, −22 were known to be associated with glioblastomas [14], [15].

Several tumor-associated lesions were also present in the BAT. Shared anomalies with ET were del(1p36), del(2p21), *MDM2* and *CDK4* amplification, amplification of 15q24.1, −19 and −22. The genetic changes of +7, del(6q27q29), *EGFR* amplification, −10, del (17p13) were limited to the ET. The summary of these results is shown in Table 4.

**Table 4:** Consistent anomalies observed in ET and BAT by a-CGH.

Consistent anomalies	ET1	BAT1	ET2	BAT2	ET3	BAT3
del (1p36)	+[27645717–29259696] (40%)	+[27645717–29259696] (45%)	+[5997076–55676921] (50%)	−	+[1009416–46806902] (40%)	+[1009416–46806902] (45%)
del (2p21)	−	−	−	−	+[47450573–47510722] (64%)	+[46985724–47510722] (70%)
**+7**	−	−	+	−	+	−
**del (6q27q29)/−6**	+Complete monosomy (34%)	−	−	−	+[163542007–166844004] (52%)	−
**amp EGFR (7p11.2)**	−	−	+[54571903–55349837]	−	−	−
−**10**	−	−	+ (60%)	−	+ (80%)	−
amp CDK4 (12q14.3)	+[64510897–64589846]	+[64510897–64589846]	−	−	−	−
amp MDM2 (12q15)	+[67369376–68348402]	+[67369376–68348402]	−	−	−	−
amp 15q24.1	+*[70483070–71401705]	+[70764425–71255997]	−	−	−	−
**del (17p13)(p53)/−17**	+[505704– 40281123] (17%)	−	+Complete monosomy (20%)	−	+Complete monosomy (40%)	−
−19	+ (30%)	−	+ (19%)	−	−	+ (15%)
−22	+ (38%)	−	+ (23%)	−	+ (23%)	+ (20%)

* further amplification in ET, with respect to BAT.

In bold: anomalies limited to ETs.

In square bracket: genomic positions (according to NCBI 36 build) of the observed anomalies.

In round brackets: mosaicism degree of the observed anomaly. The percentage of abnormal cells was inferred using the formula proposed by Valli et al. That formula cannot be applied to the amplified segments (EGFR, CDK4, MDM2 and 15q24.1) since their ploidy status is unknown.

### Immunohistology

**Figure 5:**
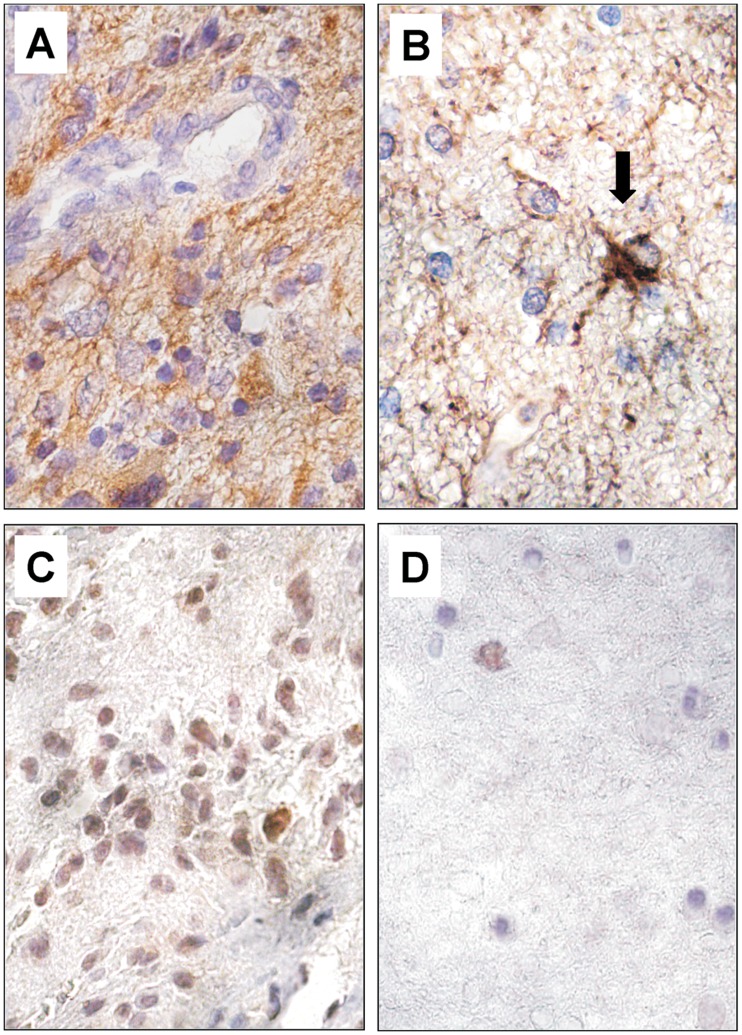
Immunohistochemical staining for GFAP and TAZ in ET and BAT. The majority of GBM cells showed intense diffuse cytoplasmic staining for GFAP (A). In the BAT, only apparently normal and reactive astrocytes (black arrow) expressed GFAP protein (B). TAZ immunoreactivity, mainly nuclear, was uniformly expressed in the cells of the ET (C). In the BAT, TAZ positive cells were observed very infrequently (D*).* Original magnification, ×630 (A–D). Hematoxylin counterstain.

**Figure 6:**
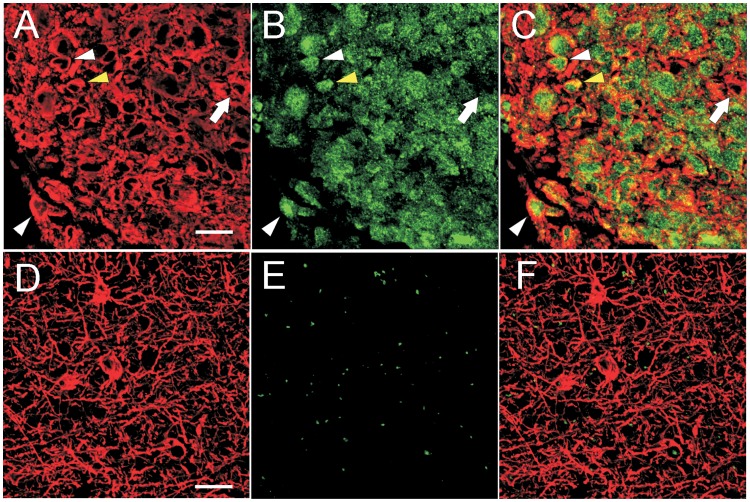
Confocal microscopy images of GFAP (red) and TAZ (green) expression in ET and BAT. In the ET samples, GFAP was highly expressed in the cytoplasm of neoplastic cells (A; C). In the BAT, it was present in the body and cytoplasmic extensions of astrocytes (D; F). In the ET, TAZ was expressed predominantly in the nucleus (white arrowheads) and few cells also showed a cytoplasmic localization (yellow arrowhead) (B; C). Rarely, the GBM cells were negative for TAZ (arrow) (B; C). In the BAT, TAZ was almost undetectable. In the photograph shown, no expression of TAZ was observed (E; F). Scale bar: 20 µm.

**Figure 7:**
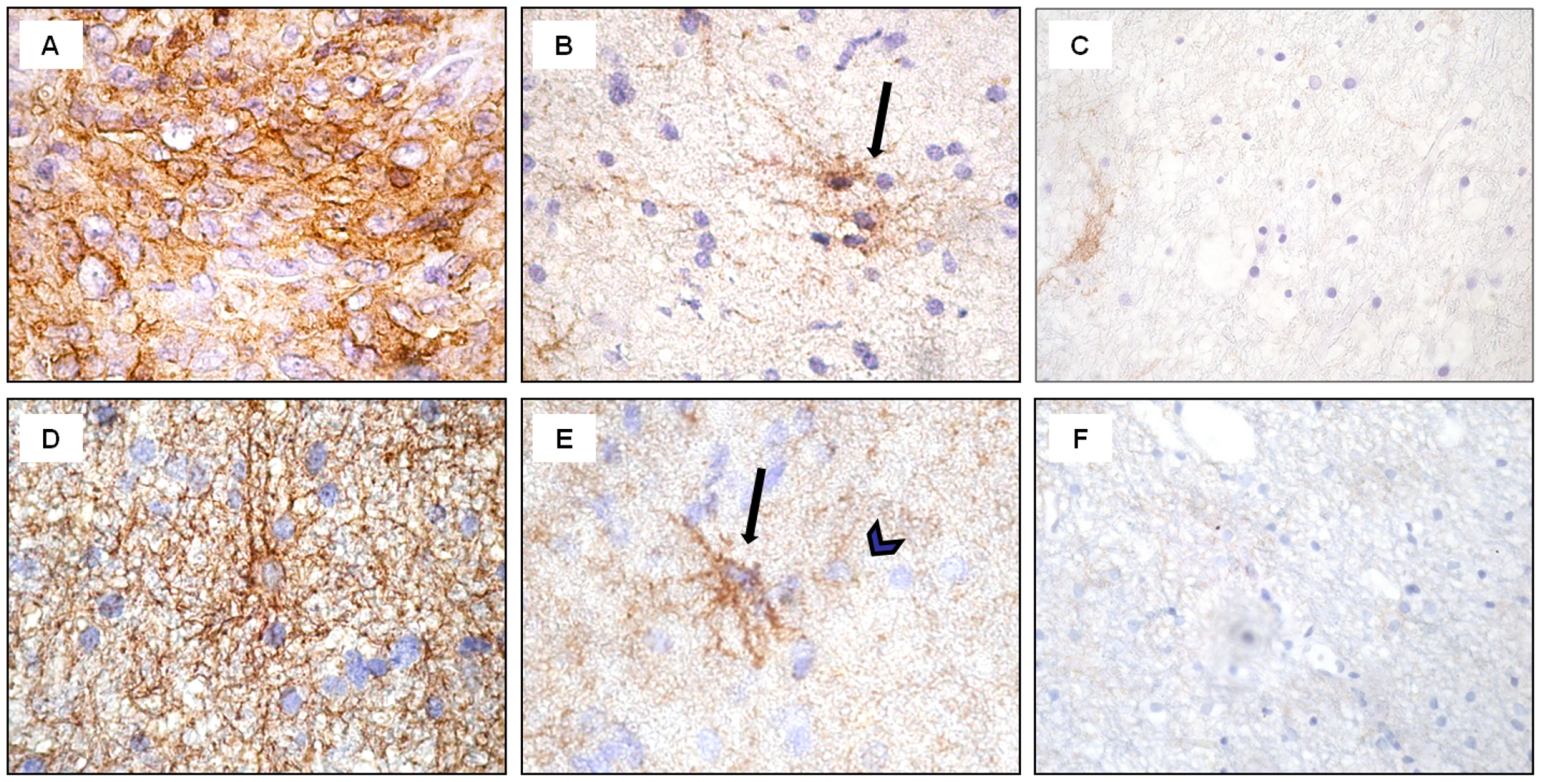
Immunohistochemical staining for EGFR and CD99 in ET, BAT and CTRL. In the ET samples, tumor cells showed intense staining for EGFR, mainly n the cell membrane (A). In the BAT, black arrow points to EGFR immunopositive reactive astrocytes (B). In the ET, CD99-immunoreactivity can be observed at the membrane level and in the cytoplasm (D). In the BAT, CD99 immunopositivity was found in reactive astrocytes (black arrow), in some normal glial cells (black arrowhead) (E). In the CTRL, immunoreactivity for EGFR or CD99 was rarely observed (C; F). Original magnification: x630 (A-B, D-E), x200 (C; F). Hematoxylin counterstain.

TAZ expression was found in the majority of GBM cells, predominantly in the cell nuclei (Figure 5C). Double-labelled ET samples revealed GFAP and TAZ co-expression when analyzed by confocal microscopy (Figure 6A–C). The majority of neoplastic cells expressed TAZ in the nucleus, and only few cells showed a cytoplasmatic staining (Figure 6B, C). As expected, GFAP was highly expressed in the cytoplasm of tumor cells (Figure 5A; Figure 6A, C). In ET samples, as expected, the majority of tumor cells exhibited strong specific EGFR immunopositivity (Figure 7A). The reactivity was strong on the cell membrane and less intense in the cytoplasm of the tumor cells. The same expression pattern was found for CD99 in the GBM cells (Figure 7D).

In BAT, GFAP immunostaining was displayed in both apparently normal and reactive astrocytes (Figure 1C; Figure 5B). The frequency of Ki67/MIB1 positive cells was always lower than 1% (Figure 1D). TAZ immunopositive nuclei were extremely rare (Figure 5D). Confocal microscopy of BAT samples confirmed that expression of TAZ was rarely seen in peritumoral tissue samples (Figure 6E, F), while apparently normal and reactive astrocytes were positive for GFAP staining (Figure 6D, F). In BAT, the EGFR immunopositivity was observed in reactive astrocytes (Figure 7B) as well as for CD99 that was also expressed in some normal cells (Figure 7E). The mean percentage of EGFR- and CD99-positive cells was higher in BAT (10.1±2.6 for EGFR and 11.5±2.8 for CD99) with respect to CTRL (3.2±0.1 and 2.7±0.2 for EGFR and CD199, respectively; Figure 7C, F).

**Figure 8:**
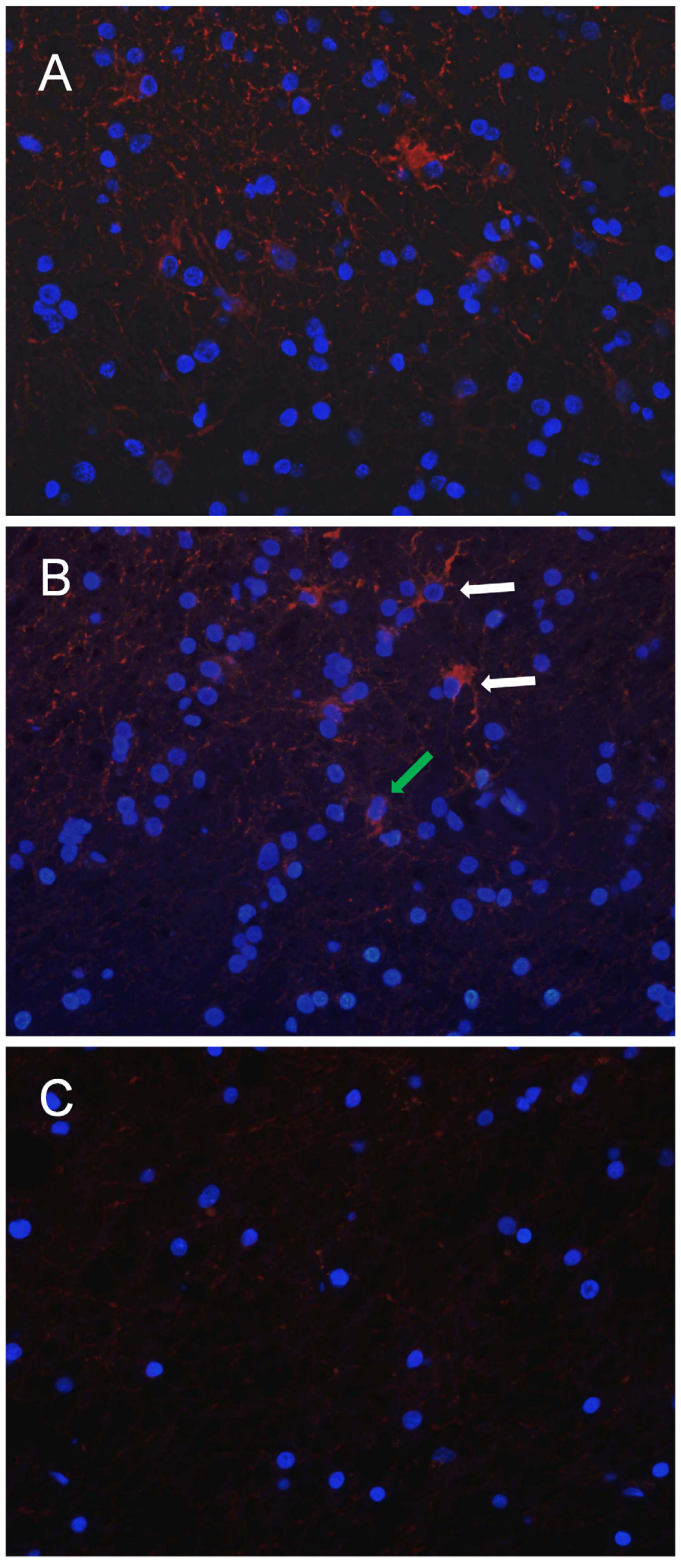
Immunofluorescence of CD133 expression in ET, BAT and CTRL. CD133 cytoplasmic immunopositivity (red) was observed in a moderate number of cells in the ET (A). In the BAT, the signal was present in reactive astrocytes (white arrows) and apparently normal cells (green arrow) (B). In CTRL samples, CD133-positive cells are extremely rare. In the field showed no CD133-positive cells are detected (C). Cell nuclei were marked with DAPI (blue). Original magnification: x400 (A, B, C).

In ET samples, CD133 cytoplasmic immunopositivity, visualized by immunofluorescence microscopy,was found in a moderate number of tumor cells (Figure 8 A). In BAT, the same pattern of reactivity was observed in a low percentage (1.9±0.3) of reactive astrocytes and apparently normal cells (Figure 8 B). A very low value (0.06±0.1) of CD133-positive cells was displayed in CTRL samples (Figure 8C).

## Discussion

Few information is available about the peritumoral tissue sampled at least one cm from the macroscopic tumor border. By comparing the expression pattern of CTRL and BAT, we separated 57 genes which were differentially expressed in BAT against CTRL. These genes were also highly expressed in GBM suggesting that GBM and morphologically normal appearing BAT share a similar expression profile. In our study, the *EGFR* expression levels showed the largest difference between CTRL and BAT, being highly expressed in the latter. The *EGFR* gene is the most frequently amplified proto-oncogene in primary glioblastomas [16]. Another growth factor receptor, *NMB*, was also overexpressed in BAT. We also found in BAT an up-regulation of *IGFBP5,* histone *HIST2H2AA* and transcription regulator *ID3.* All these elements are involved in proliferation and tumor progression [17], [18], [19].

In BAT, we also detected an over-expression of genes involved in cell motility, such as palladin, alpha-dystrobrevin (*DTNA*), *CD99* and *VCAM-1* [17], [20], [21], [22], [23], [24]. We also found the over-expression of the transcription regulator *TAZ* in BAT. This molecule controls the expression of genes regulating cell migration and proliferation [13], [25]. *TAZ* association with mesenchymal (MES) gene expression signature of glioblastoma, resulting in poor overall survival and treatment resistance has recently been emphasized [26].

The set of genes showing reduced expression in BAT included several ones previously described for their anti-oncogenic role, controlling intracellular signalling cascade and transcription molecular functions. Among these, the expression of *BAI3, PEG3, SNCA* has already been reported to be absent or significantly decreased in GBM and glioma cells [27], [28], [29].

Several genes down-regulated in BAT such as *PRDM2, TCF7L2, RB1CC1, ATP2A2* are involved in the pathogenesis of other type of cancers, but not in gliomas [30], [31], [32], [33], [34], [35]. Interestingly, *SYNJ1, NBEA, SERPINI1, CNTNAP2 and RELN,* which are known to be involved in neurogenesis, were down-regulated in BAT [36], [37], [38], [39], [40].

KLRC1, an inhibitory receptor for the non-classical MHC class I molecule HLA-E, has been involved in the inhibition of innate anti-glioma immune responses [41]. In our study, we reported the strong down-regulation of KLRC1 in BAT samples, both at transcriptional and protein level. This finding suggests that an inhibition of a proper immune response may exist. A big limitation of this study is that the “normal” white matter control samples did not come from the same patients bearing the glioblastoma, but from different patients operated for non-neoplastic lesions. The ideal would have been that the white matter controls came from the same patient but very far from the tumor (possibly in the other hemisphere). Obviously, this is not possible in vivo, due to ethical problems, and is not feasible post mortem, because at that point, the spread of the disease possibly involves multiple areas of the brain [42].

The differences between CTRL and BAT and the similarity of gene expression of BAT and GBM could be explained by the presence of infiltrative tumor cells that we were not able to detect at histological analysis. On this way, we performed the aCGH, a technique which is able to detect chromosome alterations only if these are present in a high percentage of cells. By comparing individual ET and BAT, we observed that BAT of two patients (BAT1 and BAT3) showed that almost all cells displayed anomalies consistently associated with GBM, but none of the cells of BAT2 displayed chromosomic anomalies known to be associated with GBM, even in the presence of several dysregulated genes. However, from a clinical point of view, the outcome of the patient whose BAT did not display any evident genomic change was similar to that of the other two patients. Obviously, in view of the small number of patients in the study, no conclusions on the relationship between genomic alterations of the BAT and survival can be drawn.

Overall, data interpretation is not easy. We can exclude that the differences in genes expression between CTRL and BAT reflect reactive changes as we have found the same level of gliosis, as determined by the presence of GFAP positive reactive astrocytes and the poor macrophages infiltration (data not shown). Undoubtedly, peritumor tissue sampled at 1 cm or more from the macroscopic tumor border presents frank gene/chromosome alterations. Some of these alterations were also present at the protein level, as shown by immunohistological analysis.

As a matter of fact, the mean percentage of EGFR- and CD99-positive cells in BAT was higher than 10%, while CD133 cytoplasmic immunopositivity was observed in a very low percentage (less than 2%) of reactive astrocytes and apparently normal cells. As CD133 is a marker of brain cancer stem cells, our data revealed that the amount of possible CD133 positive cancer stem cells was low in the BAT. Nonetheless, considerable experimental evidence for the existence of both CD133 positive and CD133 negative populations as tumor-initiating cells exists [43]. Therefore, we may have missed some putative cancer cells.

Undoubtedly, the array-based gene expression profile could result from a minority of cells, but this condition is in contrast with the array CGH results, showing chromosomal aberrations in the vast majority of cells.

On the other hand, the correlation between gene overexpression and encoded protein levels can also be weak.

All our experiments suggest that several tumor-like alterations are detectable in the BAT. Nevertheless, question remains about the nature of the cells populating the peritumour tissue. Two main hypotheses may support these findings and these hypotheses are not mutually exclusive. Our data could be explained by a dilution of genes expressed from tumor cells infiltrating the BAT samples: in fact the genes overexpressed in BAT against CTRL were also over-expressed in GBM but with a higher fold change. In fact, histological analysis may have missed the presence of some infiltrative tumor cells in the BAT. These cells could also be histologically undetectable, “dormant” infiltrating cells. [44].

A possible role of cancer stem cells in determining genetic changes in the BAT is difficult to sustain, since we observed few CD133 positive cells in this area, but we may have missed CD133 negative cancer stem cells.

Alternatively, these findings could be supported by a relevant amount of cells that present a gene profile compatible with a precancerous state. The aCGH data seem to be in favour of this hypothesis. Of course, the truth may lie somewhere in between, and the recurrent tumor may arise from both infiltrating tumor cells (including histologically undetectable tumor cells, “dormant” tumor cells and possibly cancer stem cells) and from an interaction and recruitment of apparently normal cells in the peritumor tissue by infiltrating tumor cells. If the latter two hypotheses are true, our observations could reflect a change of the BAT, which may progress to tumor by acquiring genomic alterations characteristic of GBM. Some changes might be induced by the cross-talk between tumor cells and normal cells. A possible mechanism of this phenomenon can be explained through the transporting of microvesicles containing RNA and a number of mRNA transcripts, which may lead to induction of oncogenic processes in peritumoral normal cells [45]. BAT may also bear traces of the tumor microenvironment in the form of microvescicles or exosomes transporting tumor mRNA or even genomic DNA, which could also partially explain our findings.

Whatever the correct explanation of our findings, further studies on the BAT are needed, in order to identify possible targets for future treatments.
